# DCZ19931, a novel multi-targeting kinase inhibitor, inhibits ocular neovascularization

**DOI:** 10.1038/s41598-022-25811-0

**Published:** 2022-12-13

**Authors:** Huiying Zhang, Bo Li, Jingjuan Ding, Rong Ye, Zhijian Xu, Qiuyang Zhang, Siguo Feng, Qin Jiang, Weiliang Zhu, Biao Yan

**Affiliations:** 1 The Affiliated Eye Hospital, Nanjing Medical University, Nanjing, China; 2 State Key Laboratory of Drug Research, Shanghai, China; 3 Drug Discovery and Design Center, Shanghai Institute of Materia Medica, Shanghai, China; 4 Eye & ENT Hospital, State Key Laboratory of Medical Neurobiology, Fudan University, Shanghai, China; 5 Shanghai Key Laboratory of Visual Impairment and Restoration, Shanghai, China; 6 National Health Commission (NHC) Key Laboratory of Myopia, Fudan University, Shanghai, China

**Keywords:** Drug discovery, Diseases

## Abstract

Neovascularization is a prominent cause of irreversible blindness in a variety of ocular diseases. Current therapies for pathological neovascularization are concentrated on the suppression of vascular endothelial growth factors (VEGF). Despite the remarkable efficacy of anti-VEGF drugs, several problems still exist, including ocular complications and drug resistance. Thus, it is still required to design novel drugs for anti-angiogenic treatment. This study aimed to investigate the anti-angiogenic effects of a small molecule multi-target tyrosine kinase inhibitor, DCZ19931, on ocular neovascularization. The results showed that administration of DCZ19931 at the tested concentrations did not cause obvious cytotoxicity and tissue toxicity. DCZ19931 could reduce the size of choroidal neovascularization (CNV) lesions in laser-induced CNV model and suppress ocular neovascularization in oxygen-induced retinopathy (OIR) model. DCZ19931 could suppress VEGF-induced proliferation, migration, and tube formation ability of endothelial cells, exhibiting similar anti-angiogenic effects as Ranibizumab. DCZ19931 could reduce the levels of intercellular cell adhesion molecule-1 (ICAM-1) expression in vivo and in vitro. Network pharmacology prediction and western blots revealed that DCZ19931 exerted its anti-angiogenic effects through the inactivation of ERK1/2-MAPK signaling and p38-MAPK signaling. In conclusion, this study indicates that DCZ19931 is a promising drug for anti-angiogenic therapy for ocular diseases.

## Introduction

Pathological neovascularization occurs in a wide range of human diseases, including cancers, rheumatoid arthritis, and proliferative retinopathies^1^. In the eye, pathological neovascularization is a critical feature of ocular diseases, such as age-related macular degeneration (AMD), diabetic retinopathy (DR), and retinopathy of prematurity (ROP)^2^. Vascular dysfunction would affect ocular microenvironment and cause local ischemia, eventually causing hemorrhage, detachment, fibrosis, and blindness. Thus, inhibiting pathological neovascularization is an effective therapeutic strategy for improving the vision of patients^3^.

During pathological neovascularization, the balance between angiogenic stimulators and angiogenic inhibitors is often interrupted. A series of pro-angiogenic factors are highly induced in pathological neovascularization, including vascular endothelial growth factor (VEGF), platelet-derived growth factor (PDGF), and matrix metalloproteinases (MMPs)^4^. Notably, VEGF acts as a pro-angiogenic factor in endothelial cells (ECs), which plays important roles in blood-vessel formation during vascular development and abnormal growth of blood vessels in diseases^5^. Increased VEGF expression can activate ECs, contributing to EC proliferation, migration, and tube formation. During pathological neovascularization, ECs can degrade the basement membrane through the action of extracellular proteases. Then, activated ECs migrate into extracellular matrix, proliferate induced by angiogenic stimulus, and form the tubules with central lumens. Hence, intervention of VEGF activity is an efficient therapeutic strategy for pathological neovascularization^6,7^.

At present, anti-VEGF medication is still the primary and first-line modality for treating ocular vascular diseases. These drugs include anti-VEGF antibodies and vascular endothelial growth factor receptor (VEGFR) fragment domain fusion proteins. However, anti-VEGF medication still has several unsatisfactory clinical consequences. Some patients have anti-VEGF drug resistance or have no response for anti-VEGF treatment^8^. Therefore, in-depth studies for developing new drugs are still required to improve the anti-angiogenic efficiency for ocular vascular diseases^9^.

Angiogenesis signaling networks are often highly complicated. To obtain a sustained and effective clinical efficiency, it is necessary to target multiple signaling targets synchronously. Multi-target kinase inhibitors (MKIs) are a series of compounds, which can simultaneously restrain the activation of multiple signaling kinases that control cell growth, proliferation, differentiation, motility, and apoptosis. To date, several MKIs have been verified to inhibit tumor growth and tumor angiogenesis^10–12^. MKIs also show great therapeutic efficiency against metabolic and autoimmune disorders, such as myelofibrosis, rheumatoid arthritis, and chronic myeloid leukemia^13^. For example, sorafenib is a dual aryl urea multi-kinase inhibitor and the first molecule-targeted drug approved for the treatment of hepatocellular carcinoma, which exerts anti-tumor and anti-angiogenic effects via inhibiting multiple targets such as VEGFR, PDGFR-β, and other serine/threonine kinases^14^. However, there are few reports about the application of MKIs in the treatment of ocular vascular diseases. In addition, although these MKIs have been widely used, several unfavorable side effects still exists during the clinical applications of MKIs. For instance, MKI drugs often have low absorption efficiency in tumors due to poor water solubility, rapid clearance, and metabolic rate. The non-specific uptake of MKIs by normal tissues further aggravate the adverse effects^13^. In this study, we designed and synthesized a new multi-kinase inhibitor, DCZ19931, and investigated its anti-angiogenic role and anti-angiogenic mechanism in ocular vascular diseases.

## Materials and methods

### Animals and ethical statement

C57BL/6J (wild-type, WT) mice were bred at constant temperature (25 °C) with alternating 12 h light–dark cycle and had free access to standard chow and clean water. All experiments were approved by the Animal Care and Use Committee of Nanjing Medical University. The experiments were carried out in compliance with the ARRIVE guidelines (https://arriveguidelines.org) and all methods were performed according to relevant guidelines and regulations.

### Synthesis of DCZ19931

**Figure 1:**
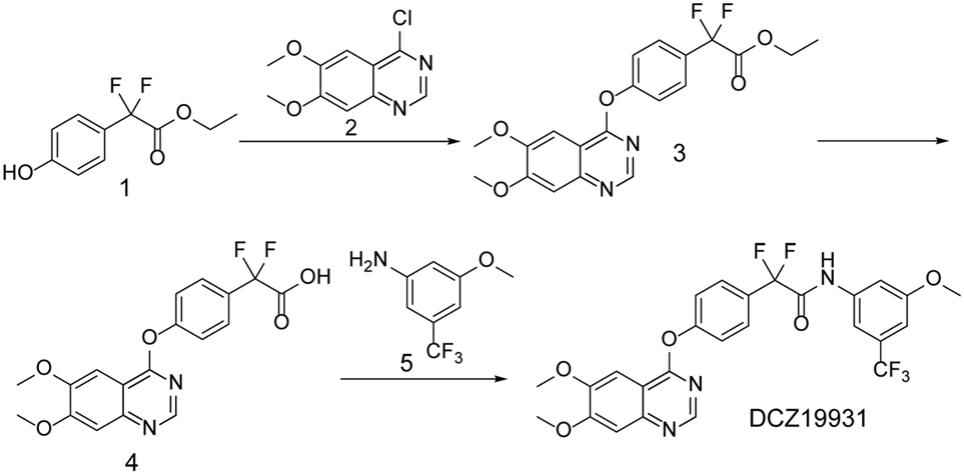
Synthesis of DCZ19931.

DCZ19931 (MW = 549.5) was designed and synthesized by Shanghai Institute of Materia Medica (Shanghai, China). The synthesis procedure of DCZ19931 was outlined in Fig. 1. Ethyl 2, 2-difluoro-2-(4-hydroxyphenyl) acetate (compound 1), 4-chloro-6,7-bis (2-methoxy) quinazoline (compound 2), triethylenediamine, and triethylamine were dissolved in acetonitrile (5 mL), followed by the reaction at 80 °C for 2 h. The organic solvent was evaporated by rotary evaporation. Compound 3 was obtained by silica gel column chromatography.

Compound 3 was dissolved in the mixture of tetrahydrofuran, methanol, and water. After the addition of 1 mol/L sodium hydroxide solution, the solution was stirred at room temperature for 2 h. The reaction solution was neutralized with 1 mol/L hydrochloric acid solution to pH 1 and extracted with ethyl acetate. The organic phases were washed with water and saturated aqueous sodium chloride solution, dried over anhydrous sodium sulfate, filtered, and evaporated to remove the solvent to produce compound 4.

Compound 4, 3-methoxy-5-(trifluoromethyl) aniline (Compound 5), N, N-diisopropylethylamine and 2-(7-azobenzotriazole)-N,N,N′,N′-tetramethylurea hexafluorophosphate were dissolved in acetonitrile and stirred at room temperature for 12 h. Then, the reaction mixture was quenched with water and extracted by ethyl acetate. The organic was separated by silica gel column chromatography to produce DCZ19931.

### Cell culture and treatment

Human umbilical vein endothelial cells (HUVECs) were obtained from American Type Culture Collection (ATCC). They were cultured in Dulbecco's modified Eagle's medium (DMEM, Gibco, C11995, USA), supplemented with 1% penicillin–streptomycin and 10% fetal bovine serum (FBS) at 37 °C with 5% CO_2_. They were pre-treated with VEGF (10 ng/mL, R&D System, 293-VE-010, USA) to mimic endothelial angiogenic effects in vitro.

### Cell viability assay

Cell viability was assessed by 3-(4, 5-dimethylthiazol-2-yl)-2, 5-diphenyltetrazolium bromide (MTT) colorimetric assay. HUVECs were treated with DCZ19931 for 24 h, and then incubated with MTT solution (5 mg/mL, BioFroxx, 1334GR001, Germany) at 37 °C for 3 h. Subsequently, isopropanol was used to dissolve the crystals. The optical density (OD) value of the absorption was detected by a microplate reader (Molecular Devices, FilterMax F5, USA).

### Flow cytometry

Annexin V-FITC/PI Apoptosis Detection Kit (Vazyme, A211-01, China) was used to detect cell apoptosis. After the required treatment, HUVECs were digested with 0.05% trypsin solution (EDTA-free) for 20 min and washed with PBS for 3 times. Subsequently, they were suspended in 200 µL binding buffer with Annexin-V-FITC (5 μL) and propidium iodide (PI, 5 μL) for 10 min in dark. Finally, cell apoptosis was detected by a flow cytometer (Beckman Coulter, CytoFLEX, USA).

### Calcein-AM/PI staining

Calcein-AM and PI double staining was conducted to examine cell apoptosis. HUVECs were seeded onto 6 well plates and cultivated in 5% CO_2_ incubator at 37 °C for 24 h. Then, they were incubated with the indicated concentrations of DCZ19931 for 24 h. HUVECs were incubated with the mixture of Calcein-AM and PI to label live cells or apoptotic cells at 37 °C for 30 min. Hoechst staining was used to label cell nuclei. The images were captured by a fluorescence microscope (Olympus, IX73, Japan) and analyzed with Image J software.

### Transwell migration assay

Cell migration ability was evaluated by the transwell migration assays. After digestion, HUVECs were re-suspended with the serum-free DMEM and added to the upper chambers of transwell (Millipore, MCEP24H48, USA) and adjusted to 5 × 10^5^/mL. Then, the lower chamber was filled with the complete media (10% FBS in DMEM). The cells were allowed to migrate for 12 h at 37 °C and 5% CO_2_. Then, the migrated cells were fixed with methanol for 15 min, washed three times with PBS for 5 min, and stained with 0.5% crystal violet for 30 min at room temperature. These non-migrated cells were gently wiped with cotton swabs. The migrated cells were counted in 6 different areas under the optical microscope (Olympus, IX73, Japan) and the average number was calculated.

### 5-Ethynyl-20-deoxyuridine (EdU) assay

EdU assays were used to detect cell proliferation by BeyoClick™ EdU Cell Proliferation Kit with Alexa Fluor 488 (Beyotime, C0071S, China). After the specific treatment, HUVECs were incubated with EdU (10 μM) for 4 h. Then, they were fixed with 4% paraformaldehyde (PFA) for 30 min, permeabilized with 0.3% Triton X-100 for 15 min, and rinsed with 3% bovine serum albumin (BSA) in PBS for 3 times. Finally, HUVECs were incubated with the click reaction solution in dark for 30 min. Cell nuclei were stained by DAPI (1:1500, Beyotime, C1002, China) at room temperature for 30 min. The images were captured by a microscope (Olympus, IX73, Japan).

### Tube formation assay

The well of 24-well plate was pre-coated with 50 μL of Matrigel basement membrane Matrix (Corning, 354234, USA) and incubated at 37 °C and 5% CO_2_ for 1 h to solidify. HUVECs were seeded onto the plated Matrigel (3 × 10^5^ cells per well) in serum-free DMEM medium and incubated at 37 °C. Images of the formation of capillary-like structures were observed after 8-h culture a computer-assisted microscope (Olympus, IX73, Japan).

### Laser-induced choroidal neovascularization (CNV) model

Laser-induced CNV models were built using C57BL/6J mice (8-week-old, male). Briefly, the mice were anesthetized with the mixture of ketamine (80 mg/kg) and xylazine (10 mg/kg). The pupils were dilated using 0.5% tropicamide. Next, four lesions surrounding the optic discs were induced by laser photocoagulation with the wavelength of 532 nm, the spot size of 50 μm, duration of 100 ms, and power of 120 mW. The lesions were induced at the 3, 6, 9, and 12 o’clock positions, which were 2–3 papillary diameters away from the optic discs. The successful disruption of Bruch’s membrane was confirmed by white bubble formation. Following laser injury, the mice were injected with 10% DMSO (2 μL, Ctrl), DCZ19931 (2 μL, 1 μg/μL), Ranibizumab (2 μL, 10 mg/mL), or 2 μL mixture solution of DCZ19931 and Ranibizumab, respectively. At day 7 after laser injury, the lesions on flat-mounted RPE/choroid were observed by immunofluorescence staining and HE staining.

### Oxygen-induced retinopathy (OIR) model

The newborn C57BL/6J mice and their nursing mothers were placed in a 75% oxygen supply chamber from P7 to P12, and then were returned to room air for 5 days. The mice received an intravitreal injection of 1 μL of 10% DMSO (Ctrl), 1 μL of DCZ19931 (1 μg/μL), 1 μL of Ranibizumab (10 mg/mL), or 1 μL mixture solution of DCZ19931 (1 μg/μL) plus Ranibizumab (10 mg/mL) immediately when returning to room air at P12. They were anesthetized and the eyeballs were removed at P17. The retinas were stained with Alexa Fluor 488-conjugated isolectin GS-IB4 (1:100, Invitrogen, I21411, USA).

### Choroidal flat-mount and isolectin-B4 staining

At day 7 following laser injury, the eyeballs were enucleated and fixed in 4% PFA for 30 min at room temperature. After the removals of cornea, lens, and retina, the eyecup was cut into 4 petals. Following washing with PBS, the flat-mount choroidal tissue was blocked with 5% BSA for 1 h at 37 °C and incubated with Isolectin-B4 (1:100, L2895, Sigma-Aldrich, USA) to label the neovascular area overnight at 4 °C. The images were photographed by a fluorescence microscope (Olympus, IX73, Japan).

### Hematoxylin and eosin (HE) staining

The histopathological change of ocular structure was observed by hematoxylin–eosin staining. Briefly, the sections were dewaxed with xylene and rehydrated with the graded concentrations of ethanol. Subsequently, the sections were successively stained with hematoxylin (1 min) and eosin (30 s). The images were taken to detect the change of ocular structures.

### Choroid sprouting assay

Choroidal explants containing RPE/choroid/sclera complexes were cut into 1 mm × 1 mm pieces. They were embedded in 40 μL of Matrigel in 24-well plates. Choroid sprouting was observed at 4 × magnification on day 4, day 5, and day 6. The sprouting area was quantified using Image J software.

### Terminal deoxynucleotidyl-transferase-mediated dUTP nick end labeling (TUNEL) staining

The apoptosis was detected by TUNEL assay using the In Situ Cell Detection Kit (Beyotime, C1088, China). After the removal of paraffin with xylene and rehydration in the graded concentrations of ethanol, the tissue sections were digested and permeated with proteinase K (20 μg/mL) for 15 min at 37 °C. Then, 50 μL of labeling reaction mixture was added to the sections for 1 h at 37 °C in dark. The sections were incubated with the recombinant DNase I (Beyotime, C1082, China) as the positive control. The nuclei were labeled with DAPI. The images were photographed by a fluorescence microscope (Olympus, IX73, Japan).

### Immunofluorescence staining

The frozen sections of the posterior eyecup were thawed and lyophilized at room temperature for 30 min and fixed with 4% PFA for 20 min. After being permeabilized and blocked with 5% BSA for 30 min, they were incubated with ICAM-1 antibody (1:200, Santa Crus, sc-107, USA) overnight at 4 °C. Then, they were washed with PBS containing 0.1% Tween 20 (PBST) three times followed by the incubation for 2 h with the secondary antibody (1:200, A11001, Invitrogen, USA). The nuclei were stained with DAPI. The images were captured by Olympus IX73.

### Cell permeability assay

Evans Blue/BSA solution (0.67 mg/mL, EBA) was prepared by diluting EB solution with BSA solution. After the required treatment, 100 μL of cell suspension was seeded in the upper chamber of transwell and 600 μL of 10% FBS was added to the lower chamber. Then, EBA solution was added to the upper chamber and the lower chamber was filled with 4% BSA solution. The OD value of absorption in the lower chamber was detected by a microplate reader (Molecular Devices, FilterMax F5, USA) after incubation for 1 h.

### Quantitative real-time polymerase chain reaction (qRT-PCR)

Total RNAs were isolated from HUVECs or retinal tissues using TRIzol (Invitrogen, 15596018, USA) following the manufacturer’s instructions. The concentration of RNA was measured according to the optical density. The integrity of RNA was estimated based on OD260/280 ratio. Total RNAs were reversely transcribed to cDNAs. qRT-PCRs were performed on PIKOREAL 96 Real-Time PCR System (Thermo Fisher Scientific, PIKOREAL 96, USA) using the PowerUp™ SYBR™ Green Master Mix (Thermo Fisher Scientific, A25742, USA). mRNA expression level of ICAM-1 was normalized to Glyceraldehyde-3-phosphate dehydrogenase (GAPDH) expression. PCR results were calculated by the relative 2^−ΔΔCt^ method.

### Potential targets intersection

In the SwissTargetPrediction database, the species was restricted to “*Homo* *sapiens*” and the candidate targets with prediction scores > 0 were selected as the potential targets of DCZ19931. For identifying the targets involved in ocular diseases, the keywords “ocular neovascularization” were searched in three databases including GeneCards (https://www.genecards.org/) database, NCBI Gene (https://www.ncbi.nlm.nih.gov/) database, and OMIM (https://www.omim.org/) database. The species was restricted to “*Homo* *sapiens*”. After merging the data, the disease targets were obtained from three databases. Subsequently, Venn diagram was generated using Venny 2.1 (https://bioinfogp.cnb.csic.es/tools/venny) to obtain the intersectant targets. The common drug-related targets and disease-related targets were input into Cytoscape software (version 3.6.1) to form a network diagram of the “drug- target-disease” interaction.

### Construction of PPI network

To determine the interaction between DCZ19931 and ocular neovascularization-related targets, the common targets of DCZ19931-disease were imported into STRING database (https://string-db.org/). The network confidence score ≥ 0.4 was set to construct protein–protein interaction (PPI) network with “*Homo* *sapiens*”. Then, Cytoscape software (version 3.6.1) was used to analyze PPI network based on the results of STRING. The color and size of the nodes were adjusted according to the degree value. The degree value presented the importance of the node in the network.

### Gene ontology (GO) and Kyoto Encyclopedia of Genes and Genomes (KEGG) pathway enrichment analysis

GO enrichment analysis was used for annotating gene function, which contains 3 aspects, including biological process (BP), molecular function (MF), and cellular component (CC). KEGG pathway analysis was used for predicting which pathway a particular gene was enriched, which covered the information resources such as diseases and pathways. The items with the adjusted *P* value < 0.05 were screened using the “clusterProfiler” package of R version 4.0.2 software to obtain the intersection target enrichment data. GO and KEGG enrichment results were represented by the bubble charts.

### Western blot

Total proteins were extracted from choroidal tissues or HUVECs by the Radio Immunoprecipitation Assay (RIPA) buffer (Beyotime, P0013B, China) containing the protease inhibitors (Roche, 04693132001, USA). The concentrations of total proteins were determined by BCA assays (Bio-Rad, 23227, USA). After electrophoresis on SDS-PAGE gels, the proteins were transferred onto the polyvinylidene difluoride (PVDF) membranes. PVDF membranes were blocked with 10% skim milk for 30 min at room temperature. Then, the membranes were incubated with the following primary antibodies at 4 °C overnight, including ERK1/2 (1:1000, Cell Signaling Technology, 9102, USA), p-ERK1/2 (1:1000, Cell Signaling Technology, 4370, USA), p38 (1:1000, Cell Signaling Technology, 9212, USA), p-p38 (1:1000, Cell Signaling Technology, 9215, USA), JNK (1:1000, Cell Signaling Technology, 9252, USA), p-JNK (1:1000, Cell Signaling Technology, 9251, USA), ICAM-1 (1:1000, Abcam, ab171123, USA), and GAPDH (1:1000, Cell Signaling Technology, 2118). Next, the membranes were incubated with the secondary antibody (1:1000, Beyotime, A0208 goat anti-rabbit IgG or A0216 goat anti-mouse IgG, China) for 2 h at room temperature. Finally, the signaling was visualized by an ECL detection kit (Beyotime, P0018S, China).

### Statistical analysis

All data are presented as means ± standard error of mean (SEM) of at least four independent experiments. The Student’s *t*-test and one-way analysis of variance (ANOVA) were performed to determine the differences between multiple groups. Statistical analyses were conducted using GraphPad Prism software. The statistical difference was considered at *P* < 0.05.

## Results

### DCZ19931 has no obvious cytotoxicity and tissue toxicity

**Figure 2:**
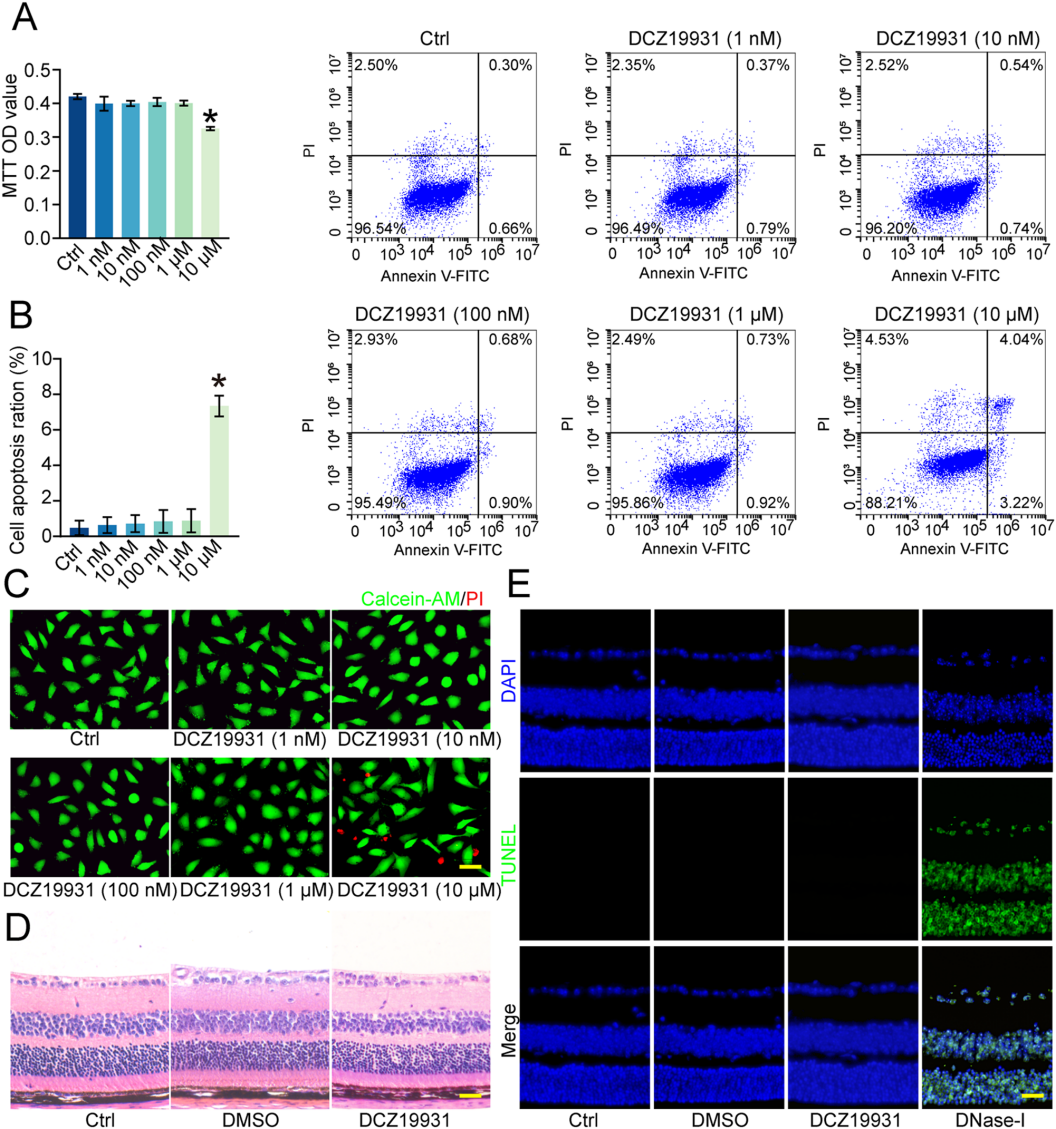
DCZ19931 has no obvious cytotoxicity and tissue toxicity. (**A**–**C**) HUVECs were incubated with the test concentrations of DCZ19931 (1 nM to 10 μM) or left untreated (Ctrl) for 24 h. Cell viability was measured by MTT assays (**A**, n = 4). Flow cytometry using Annexin V-FITC/PI double staining (**B**, n = 4) and Calcein-AM/PI staining (**C**, n = 4, scale bar, 20 μm) was performed to detect the apoptosis of HUVECs. (**D**,**E**) C57BL/6J mice received intravitreal injections of PBS (2 μL, Ctrl), 10% DMSO (2 μL), or DCZ19931 (2 μL, 1 μg/μL) for seven days. H&E staining (**D**, n = 5 animals per group, scale bar, 50 μm) and TUNEL staining assays were performed to detect retinal histological changes and retinal cell apoptosis (**E**, n = 5 animals per group, scale bar, 50 μm). DNase I was detected as a positive control in TUNEL staining assays. Statistical significance was evaluated by one-way ANOVA followed by Bonferroni post hoc test. *P < 0.05 indicated significant difference between the marked groups.

To evaluate the cytotoxicity of DCZ19931, HUVECs were treated with DCZ19931 (1 nM to 10 μM) for 24 h. MTT assay revealed that DCZ19931 had no obvious cytotoxicity on HUVECs at the test concentrations ranging from 1 nM to 1 μΜ (Fig. 2A). Calcein-AM/propidium iodide (PI) staining and Annexin V/PI staining showed that DCZ19931 did not cause a detectable apoptosis of HUVECs at the tested concentrations ranging from 1 nM to 1 μM (Fig. 2B,C).

To determine the tissue toxicity of DCZ19931, C57BL/6J mice received intravitreal injections of phosphate buffer saline (PBS, Ctrl), 10% DMSO, or DCZ19931. Hematoxylin–eosin (HE) staining indicated that DCZ19931 treatment did not cause marked histopathological changes in retinal structures (Fig. 2D). TUNEL staining demonstrated that cell apoptosis was not observed in the retinas following the administration of DCZ19931 (Fig. 2E). Taken together, these results indicates that DCZ19931 has no obvious toxicity in vitro and in vivo.

### DCZ19931 inhibits ocular neovascularization in vivo

**Figure 3:**
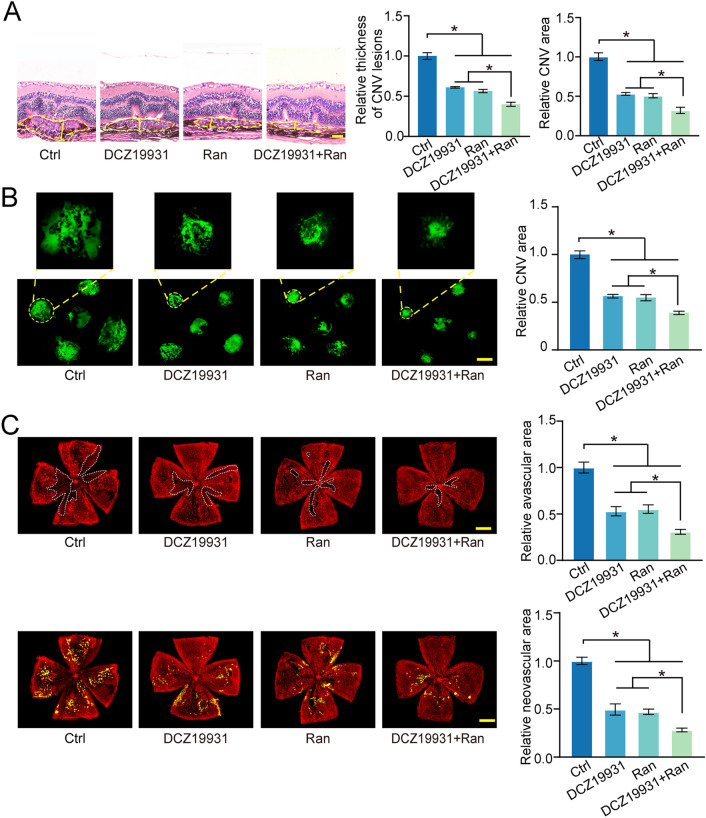
DCZ19931 inhibits ocular neovascularization in vivo. (**A**,**B**) C57BL/6J mice received intravitreal injections of 10% DMSO (2 μL, Ctrl), DCZ19931 (2 μL, 1 μg/μL), Ranibizumab (2 μL, 10 mg/mL), Ranibizumab (1 μL, 10 mg/mL) plus DCZ19931 (1 μL, 1 μg/μL) immediately after laser injury. The thickness and areas of neovascular were determined by H & E staining (**A**, n = 5, scale bar, 50 μm). CNV lesions were visualized by IB4 staining (**B**, n = 5, scale bar, 100 μm). (**C**) OIR mouse pups at P12 received intravitreal injections of 10% DMSO (1 μL, Ctrl), DCZ19931 (1 μL, 1 μg/μL), Ranibizumab (1 μL, 10 mg/mL), 1 μL of Ranibizumab (10 mg/mL) plus DCZ19931 (1 μg/μL), respectively. The retinas were harvested on P17 and then stained with GS-IB4 staining to observe retinal vessels. White dashed lines highlighted avascular areas; Yellow area indicated angiogenic regions (n = 5, scale bar, 200 μm). Statistical significance was evaluated by one-way ANOVA followed by Bonferroni post hoc test. *P < 0.05 indicated significant difference between the marked groups.

To investigate whether DCZ19931 exerted its anti-angiogenic effects in vivo, laser-induced choroidal neovascularization (CNV) model was built through the rupture Bruch’s membrane and RPE by laser photocoagulation in C57BL/6J mice. After laser injury, the mice immediately received intravitreal injections of 10% DMSO (Ctrl), DCZ19931, Ranibizumab, or DCZ19931 plus Ranibizumab. At day 7 following laser photocoagulation, the thickness and areas of CNV lesions were quantified by H&E staining (Fig. 3A). CNV regions were observed by IB4 staining (Fig. 3B). The results showed that the areas of CNV lesions were markedly reduced in DCZ19931-treated group or Ranibizumab-treated group compared with the control group. The area of CNV lesions was smallest in DCZ19931-Ranibizumab group (Fig. 3A,B).

Oxygen-induced retinopathy (OIR) model was further used to determine the anti-angiogenic effects of DCZ19931 in vivo. The pups of C57BL/6J mice at the postnatal day 7 (P7) with their nursing mothers were placed in 75% oxygen for 5 days (P12) and then returned to room air for another 5 days (P17). The mice received intravitreal injections of 10% DMSO (Ctrl), DCZ19931, Ranibizumab, or DCZ19931 plus Ranibizumab at P12. At P17, the whole‐mount retinas were visualized by GS-IB4 staining. The avascular areas were markedly reduced in DCZ19931-treated group compared with the control group. Few neovascular tufts were observed in DCZ19931-treated group. DCZ19931 plus Ranibizumab showed greater anti-angiogenic effects than DCZ19931 or Ranibizumab alone (Fig. 3C). Collectively, the above-mentioned results indicates that DCZ19931 could inhibit pathological ocular neovascularization in vivo.

### DCZ19931 exhibits anti-angiogenic effects in vitro

**Figure 4:**
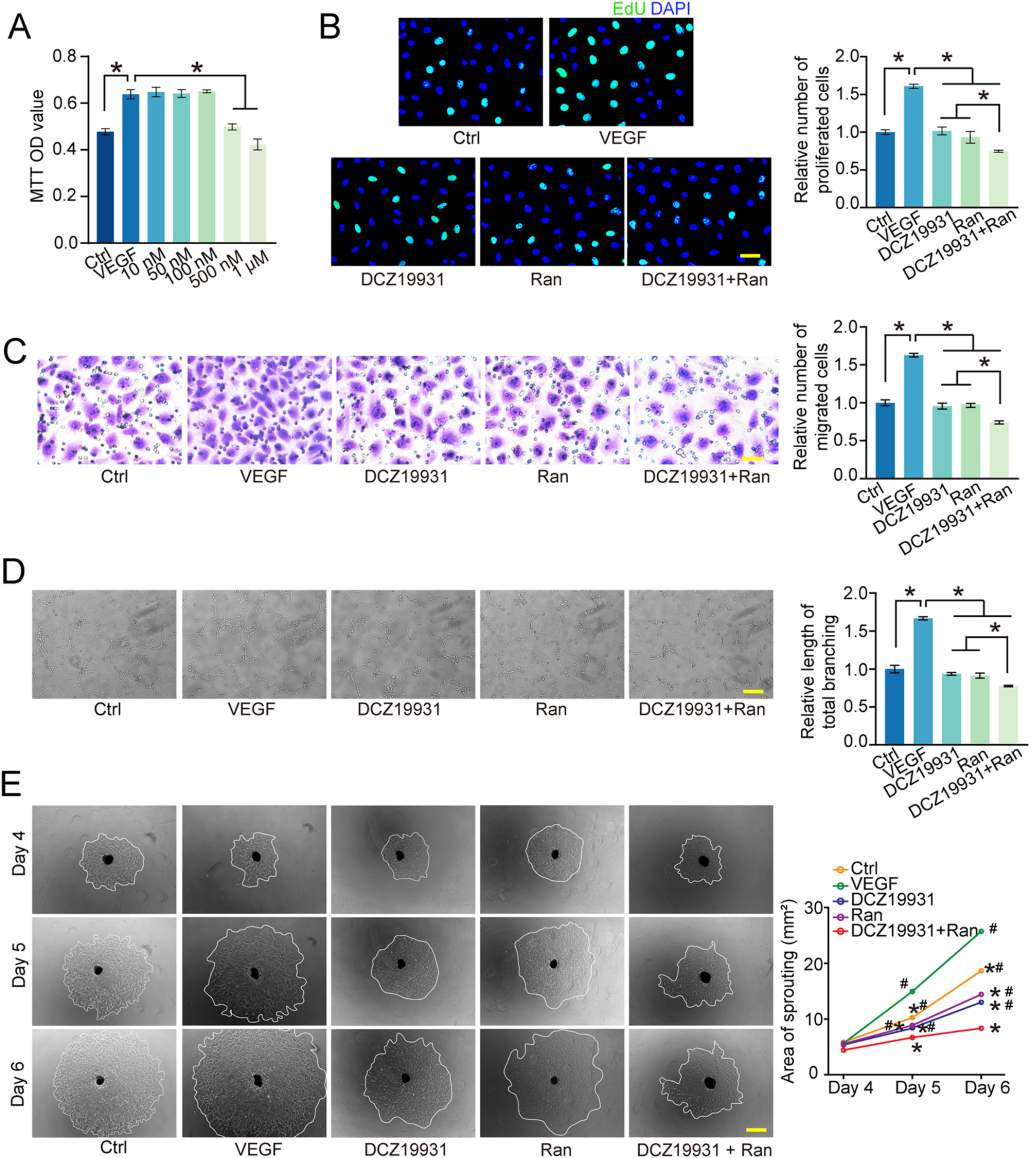
DCZ19931 exhibits anti-angiogenic effects in vitro. (**A**) HUVECs were pre-treated with VEGF (10 ng/mL) for 12 h and then treated with DCZ19931 at the concentration of 10 nM to 1 μM for 24 h. Cell viability was determined by MTT assay (n = 4). (**B**–**D**) HUVECs were pre-treated with VEGF (10 ng/mL) for 12 h and then treated with DCZ19931 (500 nM), Ranibizumab (250 μg/mL), DCZ19931 (500 nM) plus Ranibizumab (250 μg/mL), or left untreated (VEGF) for 24 h. The group without VEGF treatment was taken as Ctrl group. Cell proliferation was detected by EdU assays (**B**, n = 4, scale bar, 20 μm). The cells were allowed to migrate for 12 h at 37 °C and 5% CO_2_ and cell migration ability was assessed by transwell assays (**C**, n = 4, scale bar, 20 μm). HUVECs were seeded on matrigel matrix and cultured. Tube formation ability was observed under a light microscope at 8 h after seeding (**D**, n = 4, scale bar, 100 μm). (**E**) The mouse choroidal sprouting assays were conducted to measure the angiogenic potency of choroidal explants at day 4, day 5, and day 6 after culture. Representative images of the choroidal sprouting areas at indicated time points were shown (n = 4, scale bar, 200 µm). Statistical significance was evaluated by one-way ANOVA followed by Bonferroni post hoc test. For (**A**–**D**): *P < 0.05 indicated significant difference between the marked groups. For (**E**): *P < 0.05 vs. VEGF group, ^#^*P* < 0.05 vs. DCZ19931 + Ranibizumab group.

Endothelial cells play a critical role in pathological angiogenesis^5^. To determine whether DCZ19931 administration could regulate endothelial angiogenic effects in vitro, HUVECs were treated with VEGF (10 ng/mL) for 12 h, followed by incubation with DCZ19931 (10 nM to 1 μM) for an additional 24 h. MTT assays were carried out to detect cell viability. DCZ19931 administration could decrease cell viability induced by VEGF at the concentration of 500 nM and 1 μM (Fig. 4A). We selected 500 nM as the test concentration for the subsequent experiments. Following exposure to VEGF for 12 h, HUVECs were incubated with DCZ19931, Ranibizumab, DCZ19931 plus Ranibizumab for 24 h. The control group received no treatment. EdU assays demonstrated that DCZ19931 administration significantly decreased cell proliferation induced by VEGF (Fig. 4B). DCZ19931 administration also suppressed VEGF-mediated migration and tube formation ability of HUVECs and reduced the areas of choroidal explant sprouting (Fig. 4C–E). DCZ19931 plus Ranibizumab showed greater anti-angiogenic effects than DCZ19931 or Ranibizumab monotherapy on cell proliferation, migration, and tube formation ability as well as endothelial sprouting ability (Fig. 4B–E).

### DCZ19931 inhibits vascular permeability via downregulation of ICAM-1 expression

The formation of pathological neovascularization is often accompanied by increased vascular permeability. Intercellular cell adhesion molecule-1 (ICAM-1) is an important adhesion molecule that affects vascular permeability by binding to specific receptors on EC surfaces^15^. Here, we examined the effects of DCZ19931 administration on ICAM-1 expression and vascular permeability.

**Figure 5:**
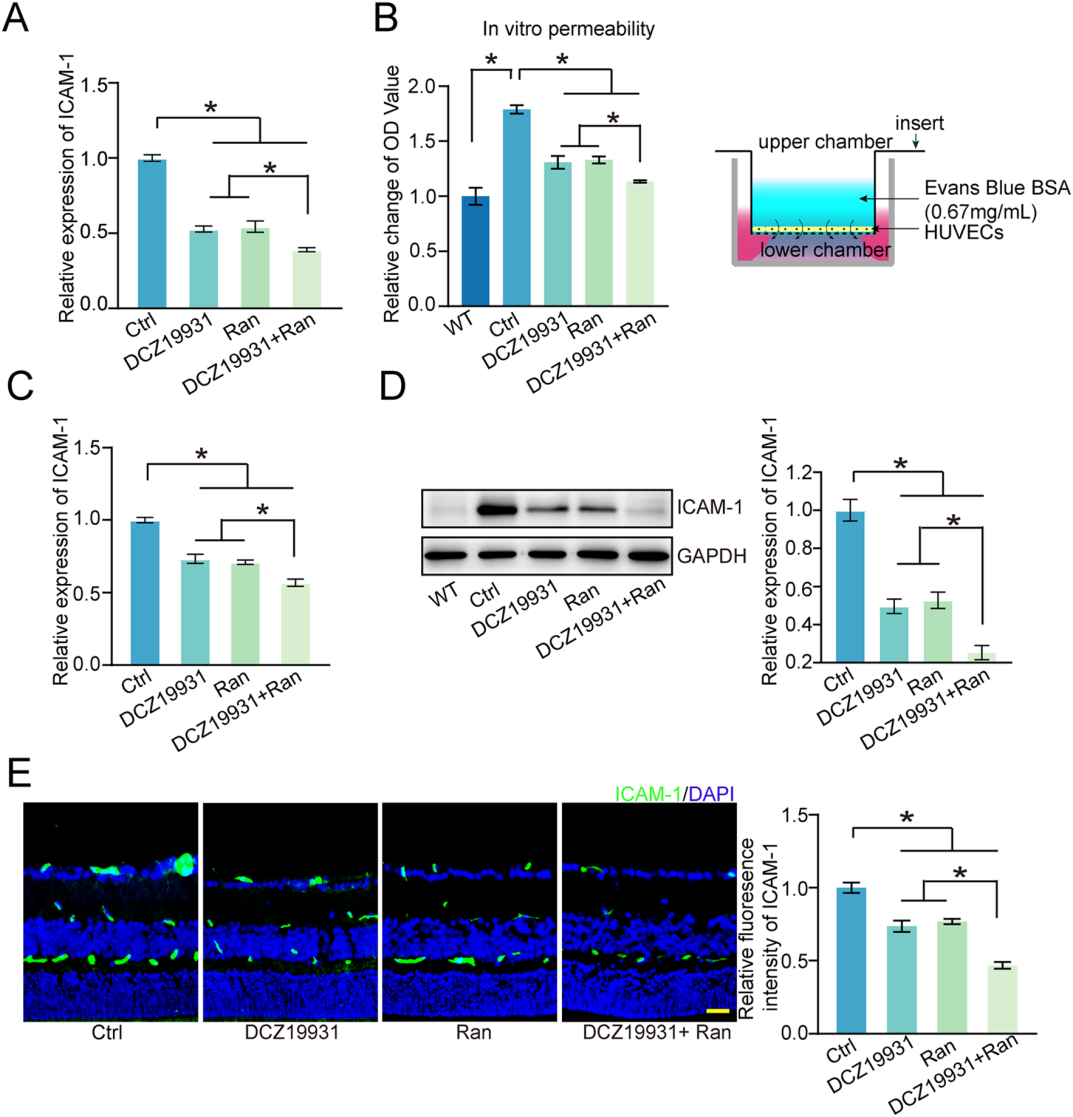
DCZ19931 inhibits vascular permeability via downregulation of ICAM-1 expression. (**A**) HUVECs were cultured with VEGF (Ctrl, 10 ng/mL), VEGF (10 ng/mL) plus DCZ19931 (500 nM), VEGF (10 ng/mL) plus Ranibizumab (250 μg/mL), VEGF (10 ng/mL) plus DCZ19931 (500 nM) and Ranibizumab (250 μg/mL) for 24 h. qRT-PCR assays were performed to detect the expression of ICAM-1 mRNA in HUVECs (n = 4). (**B**) HUVECs were cultured with VEGF (Ctrl, 10 ng/mL), VEGF (10 ng/mL) plus DCZ19931 (500 nM), VEGF (10 ng/mL) plus Ranibizumab (250 μg/mL), VEGF (10 ng/mL) plus DCZ19931 (500 nM) and Ranibizumab (250 μg/mL), or left untreated (WT) for 24 h. WT indicated normal HUVECs without VEGF induction. EB-transwell assays were conducted to detect cell permeability (n = 4). (**C**,**D**) Laser-induced CNV mice received intravitreal injections of 10% DMSO (2 μL, Ctrl), DCZ19931 (2 μL, 1 μg/μL), Ranibizumab (2 μL, 10 mg/mL), Ranibizumab (1 μL, 10 mg/mL) plus DCZ19931 (1 μL, 1 μg/μL) after laser injury. WT indicated normal mice without CNV induction. qRT-PCR assays were performed to detect the expression of ICAM-1 mRNA in choroidal tissues at day 7 after treatment (**C**, n = 5). Western blots were performed to determine ICAM-1 expression in choroidal tissues. GAPDH was used as the internal control. Representative immunoblots along with the quantitative results were shown (**D**, n = 5). (**E**) The pups of OIR mice at P12 received intravitreal injections of 10% DMSO (1 μL, Ctrl), DCZ19931 (1 μL, 1 μg/μL), Ranibizumab (1 μL, 10 mg/mL), 1 μL of Ranibizumab (10 mg/mL) plus DCZ19931 (1 μg/μL) mixture. ICAM-1 immunofluorescence assays were conducted to determine ICAM-1 expression in OIR model. The representative images with the quantitative results were shown (n = 5, scale bar, 50 μm; nuclei, blue; ICAM-1-positive cells, green). Statistical significance was determined by one-way ANOVA followed by Bonferroni post hoc test. *P < 0.05 indicated significant difference between the marked groups.

We first carried out qRT-PCR assays to detect the expression of ICAM-1 mRNA in vitro. DCZ19931 administration markedly down-regulated the levels of ICAM-1 mRNA induced by VEGF treatment in HUVECs (Fig. 5A). Cell permeability assays were performed to determine the effects of DCZ19931 administration on the barrier integrity of HUVECs. Compared with the normal group (WT group), VEGF treatment led to increased cell leakage, while DCZ19931 administration reduced cell leakage induced by VEGF treatment (Fig. 5B). Subsequently, we employed laser-induced CNV model and OIR model to investigate the effects DCZ19931 administration on ICAM-1 expression in vivo. qRT-PCR assays and western blot assays revealed that compared with normal mice (WT group), CNV induction led to up-regulated levels of ICAM-1 expression in choroidal tissues. DCZ19931 administration markedly reduced the levels of ICAM-1 expression in laser-induced CNV model (Fig. 5C,D). Similar results were observed in OIR model. Compared with OIR group (Ctrl group), the levels of ICAM-1 expression were reduced in the OIR groups treated with DCZ19931 (Fig. 5E). Moreover, the anti-angiogenic effects of DCZ19931 plus Ranibizumab treatment were greater than that of single treatment in both CNV model and OIR model (Fig. 5C–E).

### DCZ19931 inhibits ocular neovascularization through p38-MAPK and ERK1/2-MAPK signaling

**Figure 6:**
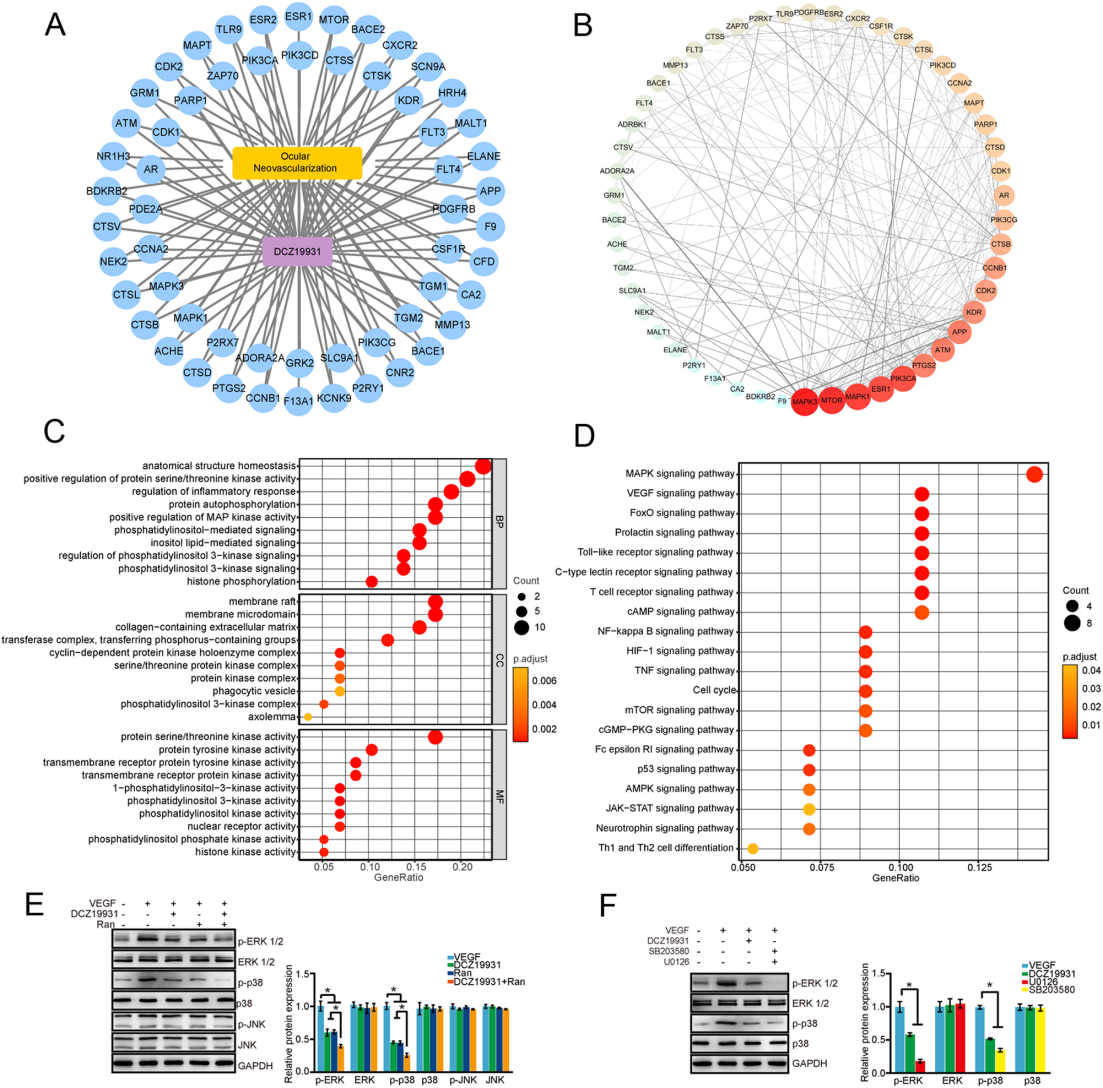
DCZ19931 inhibits ocular neovascularization through p38-MAPK and ERK1/2-MAPK signaling. (**A**) The drug-target-disease network diagram of DCZ19931 involved in ocular neovascularization. (**B**) PPI network analysis. (**C**) GO enrichment analysis. (**D**) KEGG pathways enrichment analysis. (**E**) HUVECs were treated with DCZ19931 (500 nM), Ranibizumab (250 μg/mL), DCZ19931 (500 nM) plus Ranibizumab (250 μg/mL), or left untreated (Ctrl). Then, HUVECs were stimulated with or without VEGF (50 ng/mL) for 30 min. The expression levels of total ERK1/2, p38, JNK, p-ERK1/2, p-p38, and p-JNK were detected by western blots (n = 4). (**F**) HUVECs were treated with DCZ19931 (500 nM) or SB203580 (5 μmol/mL) plus U0126 (5 μmol/mL) for 24 h, and then stimulated with VEGF (50 ng/mL) for 30 min. The group without any treatment was taken as the control (Ctrl). The phosphorylated levels of ERK1/2 and p38 proteins were detected by western blots (n = 4). GAPDH was detected as the internal control. Representative immunoblots along with the densitometric quantitative results were shown. Statistical significance was determined by one-way ANOVA followed by Bonferroni post hoc test. *P < 0.05 indicated significant difference between the marked groups.

We used the SwissTargetPrediction database to predict the targets of DCZ19931. The result showed that a total of 100 targets of DCZ19931 were obtained (Table 1). Taking “ocular neovascularization” as the keyword, a total of 2461 targets, 59 targets, and 200 targets were retrieved from GeneCards database, NCBI database, and OMIM database, respectively. After searching, summarizing, and deleting the duplicates, a total of 2563 targets related to ocular neovascularization were obtained. Then, the targets related to DCZ19931 and ocular neovascularization were input into online Venny 2.1 (https://bioinfogp.cnb.csic.es/tools/venny), a total of 58 common targets were obtained. The network diagram of “drug-target-disease” interaction was drawn by Cytoscape software (Fig. 6A).

**Table 1:** SwissTargetPrediction analysis of the targets of DCZ19931.

Common name	Uniprot ID	ChEMBL ID	Target class	Probability*
CSF1R	P07333	CHEMBL1844	Kinase	0.16565871
PDGFRB	P09619	CHEMBL1913	Kinase	0.16565871
FLT4	P35916	CHEMBL1955	Kinase	0.16565871
FLT3	P36888	CHEMBL1974	Kinase	0.148169402
KDR	P35968	CHEMBL279	Kinase	0.095623787
CTSK	P43235	CHEMBL268	Protease	0.095623787
CTSS	P25774	CHEMBL2954	Protease	0.095623787
PLA2G7	Q13093	CHEMBL3514	Enzyme	0.095623787
GCGR	P47871	CHEMBL1985	Family B G protein-coupled receptor	0.095623787
PLK1	P53350	CHEMBL3024	Kinase	0.095623787
PDK1	Q15118	CHEMBL4766	Kinase	0.095623787
SLC5A1	P13866	CHEMBL4979	Electrochemical transporter	0.095623787
PIK3CD	O00329	CHEMBL3130	Enzyme	0.095623787
PIK3CB	P42338	CHEMBL3145	Enzyme	0.095623787
PIK3CA	P42336	CHEMBL4005	Enzyme	0.095623787
ZAP70	P43403	CHEMBL2803	Kinase	0.095623787
GABRA5	P31644	CHEMBL5112	Ligand-gated ion channel	0.095623787
SCN10A	Q9Y5Y9	CHEMBL5451	Voltage-gated ion channel	0.095623787
PARP1	P09874	CHEMBL3105	Enzyme	0.095623787
CDK1/CCNB1	P06493/P14635	CHEMBL1907602	Other cytosolic protein	0.095623787
CCNE1/CDK2	P24864/P24941	CHEMBL1907605	Kinase	0.095623787
CCNE1/CDK3	P24864/Q00526	CHEMBL3038471	Kinase	0.095623787
GLRA1	P23415	CHEMBL5845	Ligand-gated ion channel	0.095623787
AR	P10275	CHEMBL1871	Nuclear receptor	0.095623787
PDE2A	O00408	CHEMBL2652	Phosphodiesterase	0.095623787
CSNK1A1	P48729	CHEMBL2793	Kinase	0.095623787
CCNA2/CDK2	P20248/P24941	CHEMBL3038469	Kinase	0.095623787
HTR7	P34969	CHEMBL3155	Family A G protein-coupled receptor	0.095623787
MAPK3	P27361	CHEMBL3385	Kinase	0.095623787
HTR5A	P47898	CHEMBL3426	Family A G protein-coupled receptor	0.095623787
MAPK1	P28482	CHEMBL4040	Kinase	0.095623787
MYLK3	Q32MK0	CHEMBL4627	Kinase	0.095623787
P2RX7	Q99572	CHEMBL4805	Ligand-gated ion channel	0.095623787
NPBWR1	P48145	CHEMBL1293293	Family A G protein-coupled receptor	0.095623787
DGAT1	O75907	CHEMBL6009	Enzyme	0.095623787
ADORA2A	P29274	CHEMBL251	Family A G protein-coupled receptor	0.095623787
ADORA2B	P29275	CHEMBL255	Family A G protein-coupled receptor	0.095623787
CETP	P11597	CHEMBL3572	Other ion channel	0.095623787
GRK2	P25098	CHEMBL4079	Kinase	0.095623787
GRM2	Q14416	CHEMBL5137	Family C G protein-coupled receptor	0.095623787
SCD	O00767	CHEMBL5555	Enzyme	0.095623787
SLC9A1	P19634	CHEMBL2781	Electrochemical transporter	0.095623787
IKBKE	Q14164	CHEMBL3529	Kinase	0.095623787
PIK3CG	P48736	CHEMBL3267	Enzyme	0.095623787
CRACR2A	Q9BSW2	CHEMBL3638353	Unclassified protein	0.095623787
TGM2	P21980	CHEMBL2730	Enzyme	0.095623787
TGM1	P22735	CHEMBL2810	Enzyme	0.095623787
S1PR1	P21453	CHEMBL4333	Family A G protein-coupled receptor	0.095623787
F13A1	P00488	CHEMBL4530	Aminoacyltransferase	0.095623787
MKNK2	Q9HBH9	CHEMBL4204	Kinase	0.095623787
MKNK1	Q9BUB5	CHEMBL4718	Kinase	0.095623787
KCNK3	O14649	CHEMBL2321613	Voltage-gated ion channel	0.095623787
KCNK9	Q9NPC2	CHEMBL2321614	Voltage-gated ion channel	0.095623787
P2RY1	P47900	CHEMBL4315	Family A G protein-coupled receptor	0.095623787
CNR2	P34972	CHEMBL253	Family A G protein-coupled receptor	0.095623787
HCRTR2	O43614	CHEMBL4792	Family A G protein-coupled receptor	0.095623787
BACE1	P56817	CHEMBL4822	Protease	0.095623787
HCRTR1	O43613	CHEMBL5113	Family A G protein-coupled receptor	0.095623787
MMP13	P45452	CHEMBL280	Protease	0.095623787
TDP1	Q9NUW8	CHEMBL1075138	Enzyme	0.095623787
CA2	P00918	CHEMBL205	Lyase	0.095623787
CFD	P00746	CHEMBL2176771	Protease	0.095623787
CA1	P00915	CHEMBL261	Lyase	0.095623787
CA12	O43570	CHEMBL3242	Lyase	0.095623787
CA9	Q16790	CHEMBL3594	Lyase	0.095623787
F9	P00740	CHEMBL2016	Protease	0.095623787
APP	P05067	CHEMBL2487	Membrane receptor	0.095623787
ELANE	P08246	CHEMBL248	Protease	0.095623787
MALT1	Q9UDY8	CHEMBL3632452	Hydrolase	0.095623787
HRH4	Q9H3N8	CHEMBL3759	Family A G protein-coupled receptor	0.095623787
SCN9A	Q15858	CHEMBL4296	Voltage-gated ion channel	0.095623787
ELOVL6	Q9H5J4	CHEMBL5704	Enzyme	0.095623787
CXCR2	P25025	CHEMBL2434	Family A G protein-coupled receptor	0.095623787
BACE2	Q9Y5Z0	CHEMBL2525	Protease	0.095623787
MTOR	P42345	CHEMBL2842	Kinase	0.095623787
CALCRL	Q16602	CHEMBL3798	Family B G protein-coupled receptor	0.095623787
ESR1	P03372	CHEMBL206	Nuclear receptor	0.095623787
ESR2	Q92731	CHEMBL242	Nuclear receptor	0.095623787
TLR9	Q9NR96	CHEMBL5804	Toll-like and Il-1 receptors	0.095623787
MAPT	P10636	CHEMBL1293224	Unclassified protein	0.095623787
CDK2/CCNA1/CCNA2	P24941/P78396/P20248	CHEMBL2094128	Other cytosolic protein	0.095623787
GRM1	Q13255	CHEMBL3772	Family C G protein-coupled receptor	0.095623787
DHODH	Q02127	CHEMBL1966	Oxidoreductase	0.095623787
ATM	Q13315	CHEMBL3797	Kinase	0.095623787
RORC	P51449	CHEMBL1741186	Nuclear receptor	0.095623787
GRIN2A/GRIN1	Q12879/Q05586	CHEMBL1907604	Ligand-gated ion channel	0.095623787
SLC6A9	P48067	CHEMBL2337	Electrochemical transporter	0.095623787
NR1H3	Q13133	CHEMBL2808	Nuclear receptor	0.095623787
BDKRB2	P30411	CHEMBL3157	Family A G protein-coupled receptor	0.095623787
PI4KB	Q9UBF8	CHEMBL3268	Enzyme	0.095623787
CTSV	O60911	CHEMBL3272	Protease	0.095623787
NEK2	P51955	CHEMBL3835	Kinase	0.095623787
CTSL	P07711	CHEMBL3837	Protease	0.095623787
GRIA2	P42262	CHEMBL4016	Ligand-gated ion channel	0.095623787
CTSB	P07858	CHEMBL4072	Protease	0.095623787
NR1H2	P55055	CHEMBL4093	Nuclear receptor	0.095623787
ACHE	P22303	CHEMBL220	Hydrolase	0.095623787
CTSD	P07339	CHEMBL2581	Protease	0.095623787
NPY5R	Q15761	CHEMBL4561	Family A G protein-coupled receptor	0.095623787
PTGS2	P35354	CHEMBL230	Oxidoreductase	0.095623787

The protein interaction network was obtained through STRING database and imported into Cytoscape for further analysis. There were 58 nodes with 211 edges in the network and the protein interaction was enriched (*P* < 0.001). The average local clustering coefficient was 0.448 and the average node degree was 7.62. The importance of nodes in the network was reflected as degree value. The higher the degree value, the more important the node in the PPI network. The top 10 key targets were MAPK3, MTOR, MAPK1, ESR1, PI3KCA, PTGS2, ATM, APP, KDR, and CDK2 ranked by the degree value (Fig. 6B).

GO terms included biological process (BP), cellular composition (CC), and molecular function (MF). The overlapping genes were enriched into 54 MF, 965 BP, and 51 CC (Fig. 6C). To determine the potential pathways of DCZ19931 involved in ocular neovascularization, a total of 121 signal pathways were predicted by KEGG enrichment analysis. The results were displayed in a bubble diagram (Fig. 6D). These targets were highly enriched in MAPK signaling pathway, VEGF signaling pathway, mTOR signaling pathway, and HIF-1 signaling pathway. Notably, MAPK signaling was ranked as the Top 1 signaling pathway involved in ocular neovascularization. We thus examined the effects of DCZ19931 administration on MAPK signaling activation.

HUVECs were cultured with DCZ19931, Ranibizumab, DCZ19931 plus Ranibizumab for 24 h. Subsequently, HUVECs were stimulated with VEGF for 30 min. Western blot assays demonstrated that the levels of phosphorylated ERK and phosphorylated p38 proteins were markedly decreased following DCZ19931 administration. DCZ19931 plus Ranibizumab had greater inhibitory effects than DCZ19931 or Ranibizumab alone on the expression levels of phosphorylated ERK and phosphorylated p38 (Fig. 6E). We also revealed that DCZ19931 administration could mimic the inhibitory effects of SB203580 (p38 inhibitor) and U0126 (ERK1/2 inhibitor) on MAPK signaling inactivation as shown by decreased expression of phosphorylated ERK and phosphorylated p38 (Fig. 6F). Collectively, the above-mentioned results suggest that DCZ19931 plays its anti-angiogenic role via the inactivation of p38-MAPK and ERK1/2-MAPK signaling pathway.

## Discussion

Neovascularization is a critical pathological factor in the progression of ocular diseases. Several pro-angiogenic and anti-angiogenic factors, including VEGF, PDGF, pigment epithelium derived factor (PEDF), could regulate the process of angiogenesis. The imbalance between pro-angiogenic and anti-angiogenic factors could lead to the formation of new blood vessels under pathological condition^3,16,17^. These hypoplastic vessels often undergo hemorrhages and leakage, leading to retinal structural change^18,19^. Currently, numerous clinical studies have shown that anti-VEGF agents have achieved good therapeutic effects in inhibiting pathological ocular neovascularization^20–22^. Despite the extensive clinical applications, there were still several deficiencies that have not been filled, such as frequent injection or drug resistance^23,24^. In this study, we designed and synthesized a new multi-target kinase inhibitor, DCZ19931, which could suppress ocular neovascularization by targeting endothelial angiogenic effects.

Although MKIs have been widely used, there are still some unfavorable side effects. For instance, these drugs have low absorption efficiency due to poor water solubility, rapid clearance, and metabolic rate. In addition, the non-specific uptake of MKIs by normal tissues may lead to a series of adverse effects^11,12^. Thus, it is required to develop new MKI drugs for anti-angiogenic treatment. Herein, DCZ19931 administration had no significant cytotoxic effects on HUVECs in vitro. HE staining and TUNEL staining revealed that DCZ19931 administration did not cause the structure changes of retinas and did not induce the detectable apoptosis in the retinas. Taken together, DCZ19931 has no obvious toxicity in vitro and in vivo and is a biosafety drug.

Ocular neovascularization contributes to visual impairment in several ocular diseases. Targeting pathological neovessels is an important advancement for treating ocular diseases^25–27^. DCZ19931 treatment could reduce the size of CNV lesions compared with the control group in the laser-induced CNV model. Moreover, the areas of avascular and neovascular were obviously reduced in the retinas of OIR model following DCZ19931 treatment. DCZ19931 suppressed the proliferation, tube formation, and migration of endothelial cells during the process of neovascularization. Notably, we compared the anti-angiogenic effects of DCZ19931 with the existing anti-VEGF drug. Ranibizumab is a monoclonal antibody fragment, which can bind closely to VEGF-A^8^. DCZ19931 had a similar anti-angiogenic efficiency as Ranibizumab. Importantly, DCZ19931 plus Ranibizumab had greater anti-angiogenic efficiency than Ranibizumab or DCZ19931 alone. The combined therapy may be used to solve the clinical problem of anti-VEGF treatment resistance.

ICAM-1 is a member of the immunoglobulin gene superfamily (IGSF) with low expression levels in endothelial cells under normal condition^28–30^. However, inflammatory stimuli such as interleukin-1β (IL-1β), tumor necrosis factor (TNF-α), and lipopolysaccharide, can significantly increase the expression of ICAM-1^31–33^. Abnormal VEGF expression can lead to endothelial angiogenic effects and vascular dysfunction, such as permeability and inflammation. In this study, we showed that VEGF administration could alter endothelial permeability. Endothelial permeability assays revealed that endothelial permeability was higher in VEGF-treated group compared with the normal group. By contrast, DCZ19931 treatment could reduce cell permeability induced by VEGF. Meanwhile, we found that DCZ19931 treatment could effectively inhibit ICAM-1 levels induced by VEGF in endothelial cells. Furthermore, DCZ19931 could suppress the expression levels of ICAM-1 in laser-induced CNV model and OIR model. These results indicate that DCZ19931 treatment could affect endothelial permeability and ICAM-1 expression in vitro and in vivo.

We further explored the molecular mechanism of DCZ19931 on ocular neovascularization based on the network pharmacology analysis. MAPK signaling pathway was predicted as the potential target pathway for DCZ19931 to exert its anti-angiogenic effects. MAPK signaling pathway can transduce extracellular stimulation signals into cells and nuclei through three-level kinase cascade^34–36^. It has been reported to be involved cell proliferation, growth, and apoptosis. There are three subfamilies including ERK1/2, p38, and JNK, which constitute a parallel MAPK signal pathway^37,38^. In this study, we found that DCZ19931 could inhibit ocular angiogenesis by reducing the phosphorylation levels of p38 and ERK1/2 proteins in MAPK signaling pathway (Supplementary Information).

## Conclusions

This study reveals that DCZ19931 presents its anti-angiogenic effects via reducing MAPK signaling pathway in endothelial cells. Importantly, the combined treatment via DCZ19931 plus Ranibizumab is more effective than single treatment. Therefore, DCZ19931 treatment is a promising therapeutic strategy for pathological ocular neovascularization.

## Supplementary Information

Supplementary Information.

## Data Availability

The datasets generated for this study are available on request to the corresponding authors.
