# Supplementary material for: DCZ19931, a novel multi-targeting kinase inhibitor, inhibits ocular neovascularization
Source: Sci Rep. 2022 Dec 13;12:21539. doi: 10.1038/s41598-022-25811-0 (PMC9747701; doi:10.1038/s41598-022-25811-0)
Supplement: Supplementary file 1 — Supplementary Information. [file 41598_2022_25811_MOESM1_ESM.pdf]

MS Title: *DCZ19931, a novel multi-targeting kinase inhibitor, inhibits ocular neovascularization*

Huiying Zhang[1, ⸸], Bo Li [2,3,4, ⸸], Jingjuan Ding[1, ⸸], Rong Ye[1], Zhijian Xu [2,3,4], Qiuyang Zhang[1], Siguo Feng[1], Qin Jiang[1,*], Weiliang Zhu[2,3,4,*], Biao Yan[5,6,7,*]

[1] The Affiliated Eye Hospital, Nanjing Medical University, Nanjing, China
[2] CAS Key Laboratory of Receptor Research, Shanghai, China
[3] State Key Laboratory of Drug Research, Shanghai, China
[4] Drug Discovery and Design Center, Shanghai Institute of Materia Medica, Shanghai, China
[5] Eye & ENT Hospital, Shanghai Medical College, Fudan University, Shanghai, China
[6] Shanghai Key Laboratory of Visual Impairment and Restoration, Shanghai, China
[7] National Health Commission (NHC) Key Laboratory of Myopia, Fudan University, Shanghai, China

*Corresponding author at: Eye Institute, Eye & ENT Hospital, Shanghai Medical College, Fudan University, Shanghai, China
*Corresponding author at: Drug Discovery and Design Center, Shanghai Institute of Materia Medica, Shanghai, China
*Corresponding author at: The Affiliated Eye Hospital, Nanjing Medical University, Nanjing, China
E-mail address:jiangqin710@126.com (Q. Jiang), biao.yan@fdeent.org (B. Yan), wlzhu@simm.ac.cn (WL. Zhu)

⸸ These authors contributed equally to this work.

Supplementary information

Figure 5D

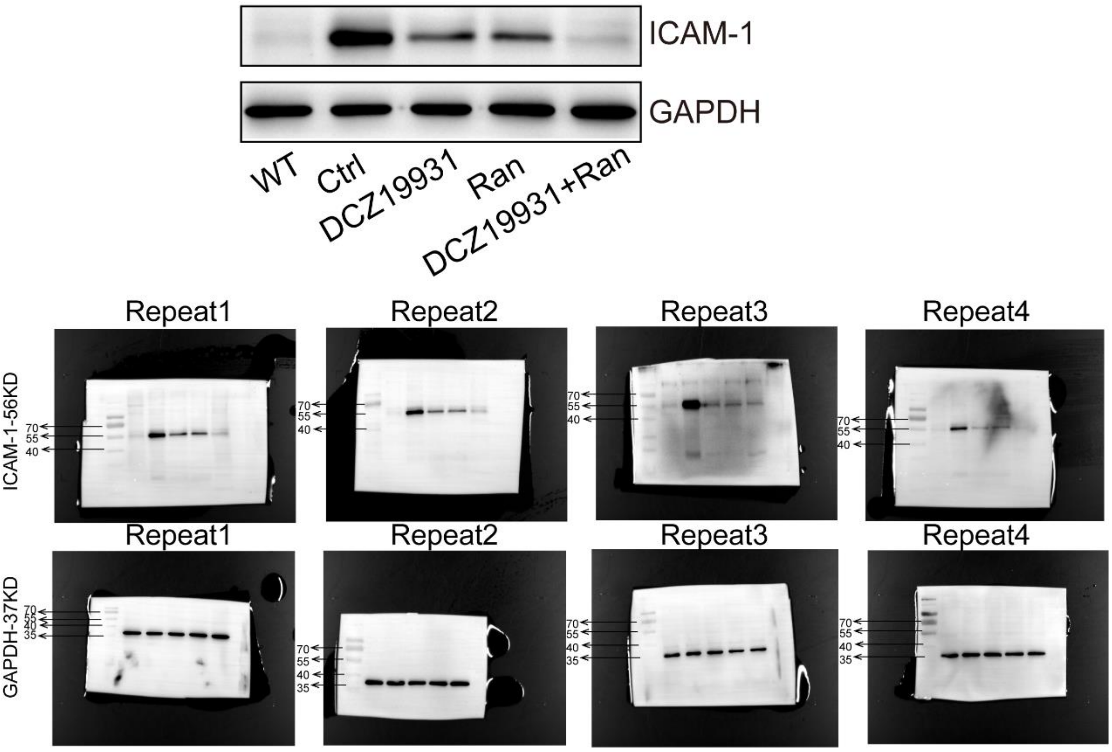

Figure 6E

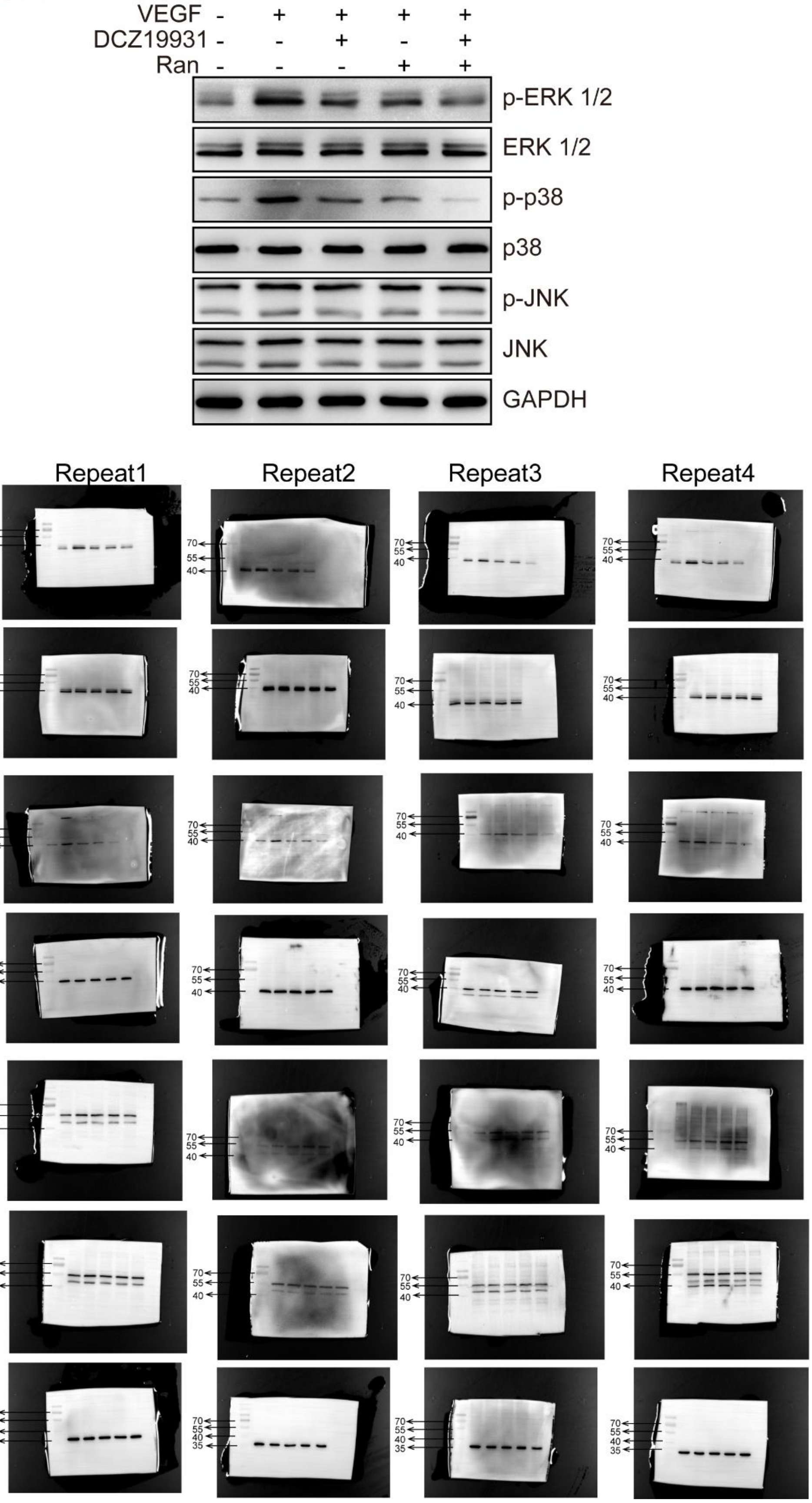
VEGF - + + + +
DCZ19931 - - + - +
Ran - - - + +
p-ERK 1/2
ERK 1/2
p-p38
p38
p-JNK
JNK
GAPDH
Repeat1
Repeat2
Repeat3
Repeat4
pERK1/2-42KD
ERK1/2-42KD
pP38-40KD
P38-40KD
PJNK-54 KD
JNK-54 KD
GAPDH-37KD
70
55
40
35

Figure 6F

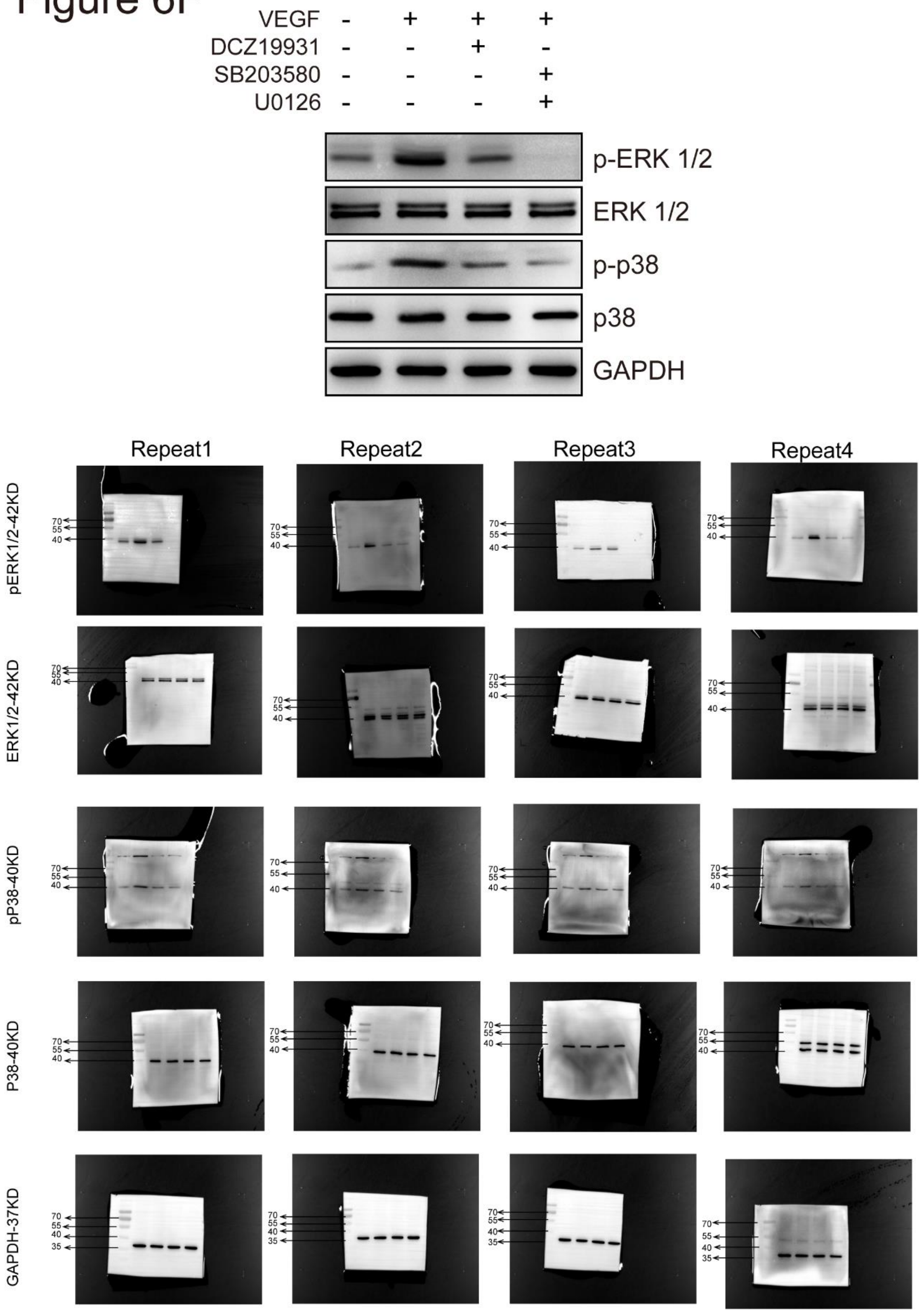
VEGF - + + +
DCZ19931 - - + -
SB203580 - - - +
U0126 - - - +
p-ERK 1/2
ERK 1/2
p-p38
p38
GAPDH
Repeat1
Repeat2
Repeat3
Repeat4
pERK1/2-42KD
ERK1/2-42KD
pP38-40KD
P38-40KD
GAPDH-37KD
70
55
40
35
